# Effects of childhood body size on breast cancer tumour characteristics

**DOI:** 10.1186/bcr2564

**Published:** 2010-04-15

**Authors:** Jingmei Li, Keith Humphreys, Louise Eriksson, Kamila Czene, Jianjun Liu, Per Hall

**Affiliations:** 1 Karolinska Institutet, Department of Medical Epidemiology and Biostatistics, Box 281, 171 77 Stockholm, Sweden; 2 Human Genetics, Genome Institute of Singapore, 60 Biopolis Street, Singapore, 138672, Singapore

## Abstract

**Introduction:**

Although a role of childhood body size in postmenopausal breast cancer risk has been established, less is known about its influence on tumour characteristics.

**Methods:**

We studied the relationships between childhood body size and tumour characteristics in a Swedish population-based case-control study consisting of 2,818 breast cancer cases and 3,111 controls. Our classification of childhood body size was derived from a nine-level somatotype. Relative risks were estimated by odds ratios with 95% confidence intervals, derived from fitting unconditional logistic regression models. Association between somatotype at age 7 and tumour characteristics were evaluated in a case-only analysis where *P* values for heterogeneity were obtained by performing one degree of freedom trend tests.

**Results:**

A large somatotype at age 7 was found to be associated with decreased postmenopausal breast cancer risk. Although strongly associated with other risk factors such as age of menarche, adult body mass index and mammographic density, somatotype at age 7 remained a significant protective factor (odds ratio (OR) comparing large to lean somatotype at age 7 = 0.73, 95% confidence interval (CI) = 0.58-0.91, *P* trend = 0.004) after adjustment. The significant protective effect was observed within all subgroups defined by estrogen receptor (ER) and progesterone receptor (PR) status, with a stronger effect for ER-negative (0.40, 95% CI = 0.21-0.75, *P* trend = 0.002), than for ER-positive (0.80, 95% CI = 0.62-1.05, *P* trend = 0.062), tumours (*P* heterogeneity = 0.046). Somatotype at age 7 was not associated with tumour size, histology, grade or the presence or absence of metastatic nodes.

**Conclusions:**

Greater body size at age 7 is associated with a decreased risk of postmenopausal breast cancer, and the associated protective effect is stronger for the ER-negative breast cancer subtype than for the ER-positive subtype.

## Introduction

There is considerable evidence that childhood anthropometric measurements are associated with postmenopausal breast cancer risk. It has been consistently shown that variables that approximate body shape and size early in life are inversely associated with breast cancer risk in adulthood. For example, a study conducted in 1998 on the same data set as used in the current study [1] reported that a larger somatotype at age seven years was associated with a lower postmenopausal breast cancer risk. Likewise, Hilakivi-Clarke and colleagues [2] found that a shorter height and higher body mass in girls from age 7 to 15 years were associated with a decreased incidence of breast cancer. Berkey and colleagues [3] also found extremely lean body mass at age 10 years to be associated with elevated breast cancer risk. In another study performed in 141,393 Danish girls, a high childhood body mass index (BMI) at age 14 years was shown to be protective against breast cancer later on in life [4]. In addition, a study performed on the large Nurses' Health Study dataset concluded that average body fatness between the ages of 5 and 10 years are inversely associated with mammographic density [5], which is generally considered to be an intermediate phenotype of breast cancer [6].

Although a role of childhood body size in adult breast cancer risk has been established, less is known about its influence on tumour characteristics. One study by Bardia and colleagues [7] looked into the risk of developing postmenopausal breast cancer stratified by estrogen receptor (ER) and progesterone receptor (PR) subtypes and reported that an increase in weight at age 12 years was associated with a decrease in adult breast cancer risk, with the most pronounced effects exhibited by ER-positive/PR-negative tumours. No significant heterogeneity, however, was observed between the tumour subtypes studied. To our knowledge, no other study has been conducted to assess whether pre-/peri-pubertal measurements of body size can also influence tumour characteristics. We thus followed up on the work of Bardia and colleagues and in the present study examined the relations between childhood body size to address if the far-reaching effects of childhood body size have any influence on tumour characteristics in adult cancers.

## Materials and methods

### Subjects

The subjects included in the current study are drawn from a population-based case-control study of postmenopausal breast cancer in Swedish-born women aged 50 to 74 years at the time of enrolment, which was between 1 October, 1993 and 31 March, 1995. Controls were randomly selected from the Swedish registry and frequency matched to the expected age distribution of the cases. Details on data collection and subjects have been described previously [1]. The final study group included 2,818 cases and 3,111 controls. Approval of the study was given by the ethical review board at the Karolinska Institutet (Stockholm, Sweden) and six other ethical review boards in the respective regions from which the subjects were based.

### Data collection and classification

**Figure 1:**
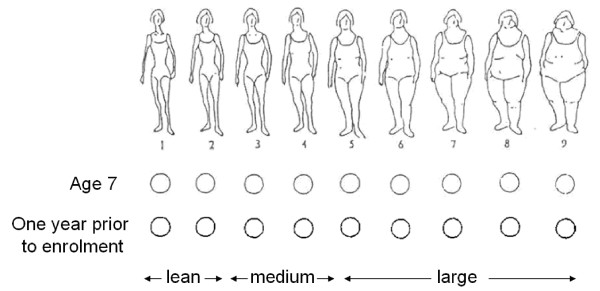
**Nine-level somatotype pictogram**.

With the exception of clinical data on tumour characteristics and mammographic density, all other covariate data were derived from the parent case-control study. Anthropometric measurements at age seven years and one year prior to enrolment were collected by means of a nine-level somatotype (Figure 1) featured in the study questionnaire, and the validity of this measurement method has been previously described [1]. These pictograms have been validated against BMI within a cohort of 100 Caucasian women from middle-class communities with an average age of 73.1 years [8]. In a population-based validation study, 111 Swedish women aged 51 to 66 years were found to have a correlation coefficient between BMI from school records and adult report of somatotype at age seven years of 0.6 [1]. The somatotypes were subsequently grouped as lean (S1 to S2), medium (S3 to S4) and large (S5 to S9) prior to analysis. Other covariate data that was collected using the self-reported study questionnaire and examined in this study include age of menarche (continuous, in years), parity (continuous, number of live births), history of benign breast disease (binary, never/ever), BMI (continuous, in kg/m^2^), history of hormone replacement therapy (HRT) (binary, never/ever), and family history of breast cancer (binary, no/yes). Age at menopause (continuous, in years) was also derived from information collected in the study questionnaire and the definition used in this study has been previously described [1]. It is defined as the age at the last menstrual period or the age at bilateral oophorectomy, if one year or more prior to data collection. Women who have had a hysterectomy, or who have not ceased menstruation due to HRT, or with missing information on age at menopause were considered to be postmenopausal if the age reported at time of questionnaire was equal to or above the 90th percentile of age at natural menopause of study subjects (current smokers: 54 years old; nonsmokers: 55 years old, independent of case/control status). Subjects classified as postmenopausal in this manner were assigned an age at menopause according to their current smoking status and the mean ages at natural menopause in our data. Otherwise, women were considered to be premenopausal and were excluded.

Information regarding the retrieval of tumour characteristics from the medical records of all participants from surgical and oncological units throughout Sweden have been presented in detail elsewhere [9,10]. The tumour characteristics in the present study included tumour size (categorical, groups in cm), grade (categorical, classified according to the Nottingham histological grade or Bloom-Richardson scale), as well as ER and PR status (binary, absent/present).

The process of collecting mammographic density data in this study has been described previously [11]. Film mammograms of the medio-lateral oblique view were digitised using an Array 2905HD Laser Film Digitizer (Array Corporation, Tokyo, Japan), which covers a range of 0 to 4.7 optical density. For controls, breast side was randomized. For cases, the side contralateral to the tumour was used. The density resolution was set at 12-bit spatial resolution. The Cumulus software used for the computer-assisted thresholding was developed at the University of Toronto [12]. For each image, a trained observer (LE) set the appropriate gray-scale threshold levels defining the edge of the breast and distinguishing dense from non-dense tissue. The software calculated the total number of pixels within the entire region of interest and within the region identified as dense. These values were used to calculate the percentage of the breast area that is dense. A random 10% of the images were included as replicates to assess the intra-observer reliability, which was high with a Spearman rank correlation coefficient of 0.95. However, as not all women attended mammographic screenings, and some mammograms were missing, such information was available for only a subset of the subjects (n = 3232, 54.5%).

### Statistical analyses

The distribution of baseline characteristics of known breast cancer risk factors were summarised as means and standard deviations or proportions. Odds ratio (OR) estimates with corresponding 95% confidence intervals (CI) were computed by fitting unconditional logistic regression models with breast cancer risk status as the response variable, adjusting for age.

To identify potential confounders of the association between somatotype at age seven years and breast cancer risk, linear/logistic regression models were fitted for either continuous (age of menarche, age of menopause, parity, BMI, and mammographic density) or binary (benign breast disease and HRT) outcomes including only controls in the analysis. Somatotype at age seven years was treated as a categorical (three-level) independent variable. Proportional odds logistic regression was used in situations where the outcome variable was ordinal (somatotypes at age seven years and one year prior to enrolment) from which cumulative OR esimates with corresponding 95% CIs were computed. Covariates were considered potential confounders if there was *a priori *evidence in the published literature of the factor being associated with both childhood body size and breast cancer risk, or if the factor was significantly associated at the 5% level with both somatotype at age seven years and breast cancer risk. Those covariates that, when added to the model, changed the coefficient by more than 10%, were considered confounders and adjusted for in the multivariate analysis. The final variables in the multivariate logistic regression model examining breast cancer risk overall, and stratified by ER and PR tumour subtypes, included age, age at menarche, benign breast disease, and BMI one year prior to enrolment (recent BMI). Adjustment for other variables did not influence the somatotype risk estimates. Mammographic density was also identified as a confounder. However, as mammographic density data are only available for a subset of the subjects, this variable was accounted for together with the other risk factors in a separate model. Women with and without mammographic density information were not found to differ significantly at the 5% level for the covariates included in the analysis models (data not shown).

Associations between somatotype at age seven years and tumour characteristics were evaluated in a case-only analysis, by fitting ordinal regression models treating tumour characteristics as dependent variables, with somatotype at age seven years included as a covariate. *P *values for heterogeneity were obtained by performing one degree of freedom trend tests. As there exists prior evidence that certain tumour characteristics such as ER status are associated with age at diagnosis [13], and that somatotype at age seven years is significantly associated with age of diagnosis at the 5% level (regression coefficient for age in years of -0.91 with corresponding 95% CI of -1.32 to -0.50), every model fitted in the case-only analysis was also adjusted for age at diagnosis. All analyses were performed using the statistical software R for Windows version 2.8.0 (R Development Core Team, Vienna, Austria) [14]. The level of significance was set at 5%. All statistical tests were two-sided.

## Results

Table S1 in Additional file 1 describes the characteristics of study subjects with respect to several breast cancer risk factors. Age of menarche was weakly but positively associated with the disease (OR per year increase in age of menarche = 0.96, 95% CI = 0.93 to 1.00, *P *= 0.057), a result consistent with the literature [4]. Family history, age at menopause, parity, age of first birth, benign breast disease, mammographic density, recent BMI and use of HRT were strongly significant for breast cancer risk with effects in a direction consistent with those estimated in other epidemiological studies. The first association analyses we performed between somatotypes at different ages and breast cancer risk were adjusted for age at enrolment only. Among the different measurements of somatotypes, only the time point at age seven years was found to affect breast cancer risk (OR per increase in somatotype class = 0.87, 95% CI = 0.8 to 0.95, *P *= 0.001). A larger proportion of cases than controls had a leaner body shape at age seven years. Despite somatotype one year prior to enrolment having a high correlation to recent BMI (Spearman correlation coefficient: 0.760, data not shown), it was not found to be significantly associated with breast cancer (OR per increase in somatotype class = 1.04, 95% CI = 0.94 to 1.15, *P *= 0.160).

To identify potential confounders of the association between somatotype at age seven years and breast cancer risk, we assessed whether other established risk factors for breast cancer are associated with somatotype at age seven years. An increase in childhood body size was found to exhibit strong inverse associations with age of menarche (OR comparing large to lean somatotype at age seven years = 0.61, 95% CI = 0.50 to 0.76, *P *trend < 0.0001), benign breast disease (0.47, 95% CI = 0.25 to 0.89, *P *trend = 0.006), and mammographic density (0.61, 95% CI = 0.41 to 0.90, *P *trend = 0.001; Table 1). Associations in the opposite direction were found for proxy measures of physique at other time points, such as birth weight (OR comparing birthweight >4000 g to ≤2500 g = 1.89, 95% CI = 0.95 to 3.76, *P *trend = 0.014), somatotype one year prior to enrolment (OR comparing large to lean somatotype at age seven years = 2.33, 95% CI = 1.70 to 3.18, *P *trend < 0.0001) and recent BMI (2.66, 95% CI = 1.47 to 4.83, *P *trend < 0.0001). No evidence of association was found between age of menopause and somatotype at age seven years or between family history and somatotype at age seven years. Parity and HRT were found to be independent of somatotype at age seven years (0.93, 95% CI = 0.76 to 1.13, *P *trend = 0.217 and 0.98, 95% CI = 0.73 to 1.32, *P *trend = 0.868, respectively).

**Table 1:** Associations of somatotype at age seven years with other breast cancer risk factors (controls only)

Risk factor (dependent variable)	Somatotype (independent variable)	n	OR	95% CI		*P *trend*
Age of menarche (years)	Lean	1456	1.00	reference		<0.0001
	Medium	669	0.72	0.64	0.82	
	Large	187	0.61	0.50	0.76	
Age of menopause (years)	Lean	1572	1.00	reference		0.697
	Medium	736	1.19	0.85	1.68	
	Large	204	0.93	0.53	1.65	
Parity (Number of live births)	Lean	1578	1.00	reference		0.217
	Medium	745	0.93	0.83	1.05	
	Large	207	0.93	0.76	1.13	
Benign breast disease	Lean	1578	1.00	reference		0.006
	Medium	745	0.76	0.56	1.03	
	Large	207	0.47	0.25	0.89	
Somatotype one year prior to enrolment	Lean	1571	1.00	reference		<0.0001
	Medium	739	1.72	1.44	2.05	
	Large	206	2.33	1.70	3.18	
BMI (kg/m^2^)	Lean	1562	1.00	reference		<0.0001
	Medium	742	1.85	1.30	2.65	
	Large	205	2.66	1.47	4.83	
Percent mammographic density (%)†	Lean	862	1.00	reference		0.001
	Medium	428	0.72	0.58	0.91	
	Large	108	0.61	0.41	0.90	
HRT	Lean	1569	1.00	reference		0.868
	Medium	739	0.99	0.83	1.18	
	Large	206	0.98	0.73	1.32	
	Other independent variables					
						
Birthweight (g) on somatotype at age 7	≤2500	49	1.00	reference		0.014
	2500-3000	229	1.18	0.61	2.29	
	3000-3500	470	1.29	0.68	2.43	
	3500-4000	397	1.44	0.76	2.73	
	>4000	135	1.89	0.95	3.76	
Family history on somatotype at age 7	No	2258	1.00	reference		0.485
	Yes	227	1.10	0.84	1.44	

* Based on Wald tests for regression coefficients in continuous, ordinal or logistic regression models (see statistical analyses section). All regression models were adjusted for age at enrolment. † Subset with phenotypic data. BMI, body mass index; CI, confidence interval; HRT, hormone replacement therapy; OR, odds ratio.

After adjustment of known breast cancer predictors and other associated risk factors, the inverse association of somatotype at age seven years with breast cancer remained highly significant (Table 2; OR comparing large to lean somatotype at age seven years = 0.73, 95% CI = 0.58 to 0.91, *P *trend = 0.004). The protective effect of a larger somatotype was found to be significant (*P *trend < 0.05) for ER-negative, PR-positive and PR-negative subtypes and marginally significant (*P *trend = 0.062) for the ER-positive subtype. Within the group consisting of large somatotypes, the most prominent effects were shown in ER-negative (OR comparing large to lean somatotype at age seven years = 0.40, 95% CI = 0.21 to 0.75, *P *trend = 0.002) and PR-negative (0.63, 95% CI = 0.40 to 0.99, *P *trend = 0.028) tumours. The point estimates changed very little before and after additional adjustment for mammographic density as a continuous variable [see Table S2 in Additional file 2], using a subset of the data with this information available (n = 3232).

**Table 2:** Multivariate-adjusted OR estimates and corresponding 95% CIs of postmenopausal breast cancer for somatotype at age seven years, overall and stratified by breast cancer tumour subtype based on ER and PR status

Type of breast cancer	Somatotype	All subjects				
		
		Cases	OR	95% CI		*P *trend*
All data	Lean	1784	1.00	reference		0.004
	Medium	757	0.90	0.79	1.02	
	Large	173	0.73	0.58	0.91	
ER positive	Lean	963	1.00	reference		0.062
	Medium	408	0.91	0.78	1.06	
	Large	98	0.80	0.62	1.05	
ER negative	Lean	219	1.00	reference		0.002
	Medium	81	0.77	0.58	1.03	
	Large	14	0.40	0.21	0.75	
PR positive	Lean	841	1.00	reference		0.027
	Medium	354	0.89	0.75	1.04	
	Large	83	0.76	0.57	1.00	
PR negative	Lean	320	1.00	reference		0.028
	Medium	126	0.86	0.68	1.08	
	Large	25	0.63	0.40	0.99	

* Logistic regression models were used, accounting for age, age at menarche, benign breast disease and recent body mass index. CI, confidence interval; ER, estrogen receptor; OR, odds ratio; PR, progesterone receptor.

We next assessed the effects of childhood body size on tumour characteristics (ER status, PR status, tumour size, grade, histology, and absence/presence of metastatic nodes) by fitting binary/ordinal logistic regression models, adjusting for age at diagnosis in years as a confounder. We established that the protective effect of somatotype at age seven years was significantly stronger for ER-negative disease than for ER-positive disease (*P *heterogeneity = 0.046; Table 3). When comparing between two extreme groups, women with a larger body size at age seven years were 1.71 times (95% CI = 0.96 to 3.06) more likely to get ER-positive than ER-negative disease after menopause. Although the estimated trend suggests that women with the same physique are more likely to get the PR-positive disease in adulthood, the difference between the two tumour subtypes was not significant (*P *heterogeneity = 0.283). The point estimates for tumour size, histology, grade, or the presence or absence of metastatic nodes did not vary much before and after adjustment for age of diagnosis as a continuous variable.

**Table 3:** Relation of somatotype at age seven years to tumour-defined characteristics of breast cancer

Tumour characteristics	Categories	Somatotype at age seven years			*P *heterogeneity§
			
		S1-S2	S3-S4	S5-S9	
Tumour size (cm)*	<1	300	138	39	
	1-2	752	299	70	
	2-3	366	152	31	
	3-4	116	66	14	
	4-5	52	16	3	
	>=5	65	26	4	
Cumulative OR (95% CI)		1.00 (ref.)	1.00 (0.85-1.17)	0.78 (0.58-1.05)	0.255
Cumulative OR (95% CI) §		1.00 (ref.)	1.00 (0.85-1.18)	0.78 (0.58-1.06)	0.266
Grade*	Low	159	69	20	
	Medium	479	186	46	
	High	463	222	51	
Cumulative OR (95% CI)		1.00 (ref.)	1.15 (0.94-1.41)	0.99 (0.69-1.43)	0.443
Cumulative OR (95% CI) §		1.00 (ref.)	1.15 (0.93-1.41)	0.99 (0.69-1.42)	0.463
Histology*	Ductal	1350	570	137	
	Lobular	206	77	16	
	All other	92	37	7	
Cumulative OR (95% CI)		1.00 (ref.)	0.91 (0.72-1.15)	0.76 (0.48-1.20)	0.192
Cumulative OR (95% CI) §		1.00 (ref.)	0.92 (0.73-1.17)	0.79 (0.50-1.25)	0.265
Metastatic nodes†	Absent	1159	473	116	
	Present	513	227	46	
OR (95% CI)		1.00 (ref.)	1.08 (0.90-1.31)	0.90 (0.63-1.28)	0.923
OR (95% CI) §		1.00 (ref.)	1.07 (0.88-1.29)	0.86 (0.60-1.23)	0.878
ER status†	Negative	219	81	14	
	Positive	963	408	98	
OR (95% CI)		1.00 (ref.)	1.15 (0.87-1.52)	1.59 (0.89-2.84)	0.089
OR (95% CI) §		1.00 (ref.)	1.18 (0.89-1.56)	1.71 (0.96-3.06)	**0.046**
PR status†	Negative	320	126	25	
	Positive	841	354	83	
OR (95% CI)		1.00 (ref.)	1.07 (0.84-1.36)	1.26 (0.79-2.01)	0.307
OR (95% CI) §		1.00 (ref.)	1.07 (0.84-1.37)	1.28 (0.80-2.03)	0.283

*Proportional odds logistic regression models were used. † Logistic regression models were used. ‡ Derived from one degree of freedom trend tests. § Adjusted for age at diagnosis. CI, confidence interval; ER, estrogen receptor; OR, odds ratio; PR, progesterone receptor.

## Discussion

Our first main finding was that a large somatotype at age seven years was associated with a decreased risk of postmenopausal breast cancer. Although strongly associated with other risk factors such as age of menarche, adult BMI and mammographic density, somatotype at age seven years remained a significant protective factor (OR comparing large to lean somatotype at age seven years = 0.73, 95% CI = 0.58 to 0.91, *P *trend = 0.004) after adjustment for these other risk factors. Our second and most novel finding was of a significant protective effect of somatotype at age seven years regardless of receptor status, but with a stronger effect for ER-negative (0.40, 95% CI = 0.21 to 0.75, *P *trend = 0.002), than for ER-positive (0.80, 95% CI = 0.62 to 1.05, *P *trend = 0.062), tumours (*P *heterogeneity = 0.046).

Our findings regarding the protective effects of childhood body size for adult breast cancer are consistent with previous studies [3-5]. Associations with other breast cancer risk factors were also in the same direction as found in other epidemiological studies. Several studies have found birth weight and gain in BMI in early childhood to predict adult lean mass, while adult adiposity has been attributed to weight gain in late childhood and adolescence [15-19]. Similarly, anthropometric measurements at other time points (birth weight, and somatotype one year prior to enrolment) in our data were found to be positively associated with somatotype at age seven years. The adverse effects of birth weight and adult body mass on postmenopausal breast cancer risk may be explained by a surplus of estrogen exposure from either the uterine environment or excess adipose tissue [4,20]. However, studies performed on children have not consistently found an association between obesity and circulating estradiol levels [21,22], thus it is unclear what mechanisms drive the associated decrease in risk during the pre-/peri-puberty window.

Strong inverse relationships found between childhood body size, age of menarche, benign breast disease, and mammographic density were in line with other reports in the literature. Baer and colleagues [23] found a large childhood body size to be associated with a decrease in risk of benign breast disease. Age of menarche is often considered along with age of menopause and other hormonal risk factors for a woman's cumulative exposure to estrogen [24,25]. An earlier age of menarche is associated with an increased risk of breast cancer. On the other hand, a larger childhood somatotype, which is associated with decreased breast cancer risk, is also associated with an earlier age of menarche. As age of menarche is an established but weak predictor of breast cancer risk, its pronounced inverse relationship with childhood body size when seen in the context of breast cancer risk seems to be counterintuitive [26,27].

Mammographic density has also been found by others to be associated with childhood body mass [5]. Estrogen is produced by adipose tissue in the body. A higher BMI is thus correlated with higher endogenous estrogen levels. In a murine study, exposure to estrogen prior to puberty led to a decrease in radiologically dense tissue and an increase in the number of radiolucent structures [28], which may be analogous to a lower mammographic density in humans. In agreement, McCormack and colleagues [29] showed that high childhood BMI was associated with a lower Wolfe grade, and Samimi and colleagues [5] found that a rounder pre-pubertal body shape was predictive of lower mammographic density later in life.

The age-adjusted case-only comparison of our data reflected a significant difference in the effects of childhood body size on the two ER subtypes (*P *trend = 0.046), but not the PR subtypes. However, in lieu of the fact that PR is an estrogen-induced target gene, and that its presence could serve to indicate ER functional capacity and tumour differentiation state [30], we also conducted stratified analyses on PR subtypes. We found that the protective trend conferred by a larger childhood somatotype on postmenopausal breast cancer applies to all ER and PR tumour subtypes. Overall our results were consistent with Bardia and colleagues [7], although in that study the effects were only significant for ER-positive (0.80, 95% CI = 0.67 to 0.96) and PR-negative (0.62, 95% CI = 0.43 to 0.89) tumours (comparing women with above average weight at age 12 years to women with average weight at age 12 years). Although Bardia and colleagues observed a stronger protective effect in ER-negative tumours than in their ER-positive counterparts (in agreement with our finding) when comparing women with above average weight at age 12 years to women with average weight at age 12 years, the association they observed in this subgroup was not statistically significant (0.77, 95% CI = 0.5 to 1.19).

Hormonal exposure and mammographic density are established risk factors of breast cancer that have been suggested to be independent, operating through different pathways [31]. Adjustment for these factors and other traditional risk factors did not attenuate the negative association of childhood body size on breast cancer risk (OR comparing large to lean somatotype at age seven years = 0.73, 95% CI = 0.58 to 0.91, *P *trend = 0.004, for association, after adjustment), thus suggesting an independent underlying mechanism. We speculate that a possible mechanism driving the negative association with breast cancer risk could be epigenetic changes that occur during mammary development. Hilakivi-Clarke [32] summarised in a review several perspectives on special windows of mammary development. Mammary tissue is postulated to undergo epigenetic extensive modelling or re-modelling during different stages in life such as fetal development, puberty or pregnancy. Such epigenetic modification can persist into adulthood if taken place in mammary stem cells, uncommitted mammary myoepithelial or luminal progenitor cells and inherited by subsequent daughter cells [33]. Prepubertal exposure to estrogen has been shown to upregulate the expression of BRCA1, a well-known DNA repair gene [28]. Liu and colleagues [34] also demonstrated that BRCA1 is responsible for differentiating ER-negative stem/progenitor cells into ER-positive luminal cells. They also proposed that loss of expression of the DNA repair gene (BRCA1) may result in an accumulation of ER-negative stem cells with multiple genetic defects. Incidentally, loss of BRCA1 is frequently associated with ER-negative breast cancers [35]. The evidence for altered gene expression possibly caused by childhood body size helps to explain the general reduction in breast cancer risk overall. The apparent differential protection conferred to the ER-negative subtype could possibly be driven by the same underlying mechanism that operates through epigenetic modifications.

The strengths of our study include it being a population-based study, its large sample size and detailed information on many variables: anthropometric measures at different time points throughout life, mammographic density, reproductive and hormonal risk factors, and tumour characteristics. To our knowledge, this is the first study to consider the effects of somatotype at age seven years on adult breast cancer with the consideration of mammographic density, and also the first to examine its effects on tumour characteristics other than ER status.

A limitation of our study is that risk factor data were self-reported, and could thus be measured with error. Although two studies have demonstrated the validity of using the nine-level somatotype diagram for the long-term recall of childhood body size via high correlations with BMI at the same ages [8,36], it is noteworthy that in those studies no woman recalled their figure as larger than level seven in these studies, and that women with large body size were more likely to misreport their childhood somatotypes than women who were lean. However, any such measurement error is most likely to attenuate any association between childhood body size and breast cancer risk [37]. In addition, as the questionnaire study was conducted post-diagnosis of breast cancer, recall bias could have been introduced. Although the nine-level somatotype measure has not been validated specifically in a group of breast cancer cases, it is unlikely that childhood body size was differentially recalled by breast cancer cases and by controls.

## Conclusions

Our findings may have important implications. The effects of childhood body size on the different breast cancer subtypes are independent of other breast cancer risk factors, such as mammographic density and estrogen exposure. Given the strength of the associations, and the ease of retrieval of information on childhood somatotypes retrospectively from pictures early in life, childhood body size is potentially useful for building breast cancer risk or prognosis prediction models. It appears counterintuitive that a large body size during childhood can reduce breast cancer risk or alter one's prognosis, because a large birth weight and a high adult BMI have been shown to otherwise elevate breast cancer risk. There remain unanswered questions on mechanisms driving this protective effect. Because body size and related hormonal exposures are modifiable risk factors, women might substantially decrease their risk of breast cancer, in particular the more aggressive ER-negative disease, by monitoring their nutrition and exogenous hormone intake at different points in life.

## Abbreviations

BMI: body mass index; CI: confidence interval; ER: estrogen receptor; HRT: hormone replacement therapy; OR: odds ratio; PR: progesterone receptor.

## Competing interests

The authors declare that they have no competing interests.

## Authors' contributions

JLi participated in the study design, carried out the analyses and drafted the manuscript. LE digitised and obtained readings for the mammograms. KH, KC, JLiu and PH participated in study design and coordination and helped to draft the manuscript. All authors read and approved the final manuscript.

## Supplementary Material

Additional file 1 **Table S1**. Descriptive characteristics of post-menopausal women. Click here for file

Additional file 2 **Table S2**. Multivariate-adjusted odds ratio (OR) estimates and corresponding 95% confidence intervals (CIs) of postmenopausal breast cancer for somatotype at age seven years on a subset of women with mammographic density data; overall and stratified by breast cancer tumour subtype based on estrogen receptor (ER) and progesterone receptor (PR) status. Click here for file

## References

[B1] Magnusson C Baron J Persson I Wolk A Bergstrom R Trichopoulos D Adami HO Body size in different periods of life and breast cancer risk in post-menopausal women Int J Cancer 1998 76 29 34 10.1002/(SICI)1097-0215(19980330)76:1<29::AID-IJC6>3.0.CO;2-# 9533758

[B2] Hilakivi-Clarke L Forsen T Eriksson JG Luoto R Tuomilehto J Osmond C Barker DJ Tallness and overweight during childhood have opposing effects on breast cancer risk Br J Cancer 2001 85 1680 1684 10.1054/bjoc.2001.2109 11742488 PMC2363976

[B3] Berkey CS Frazier AL Gardner JD Colditz GA Adolescence and breast carcinoma risk Cancer 1999 85 2400 2409 10.1002/(SICI)1097-0142(19990601)85:11<2400::AID-CNCR15>3.0.CO;2-O 10357411

[B4] Ahlgren M Melbye M Wohlfahrt J Sorensen TI Growth patterns and the risk of breast cancer in women N Engl J Med 2004 351 1619 1626 10.1056/NEJMoa040576 15483280

[B5] Samimi G Colditz GA Baer HJ Tamimi RM Measures of energy balance and mammographic density in the Nurses' Health Study Breast Cancer Res Treat 2008 109 113 122 10.1007/s10549-007-9631-7 17592770

[B6] Boyd NF Rommens JM Vogt K Lee V Hopper JL Yaffe MJ Paterson AD Mammographic breast density as an intermediate phenotype for breast cancer Lancet Oncol 2005 6 798 808 10.1016/S1470-2045(05)70390-9 16198986

[B7] Bardia A Vachon CM Olson JE Vierkant RA Wang AH Hartmann LC Sellers TA Cerhan JR Relative weight at age 12 and risk of postmenopausal breast cancer Cancer Epidemiol Biomarkers Prev 2008 17 374 378 10.1158/1055-9965.EPI-07-0389 18250344 PMC2575770

[B8] Must A Willett WC Dietz WH Remote recall of childhood height, weight, and body build by elderly subjects Am J Epidemiol 1993 138 56 64 8333427 10.1093/oxfordjournals.aje.a116777

[B9] Rosenberg LU Einarsdottir K Friman EI Wedren S Dickman PW Hall P Magnusson C Risk factors for hormone receptor-defined breast cancer in postmenopausal women Cancer Epidemiol Biomarkers Prev 2006 15 2482 2488 10.1158/1055-9965.EPI-06-0489 17164374

[B10] Orgeas CC Hall P Rosenberg LU Czene K The influence of menstrual risk factors on tumor characteristics and survival in postmenopausal breast cancer Breast Cancer Res 2008 10 R107 10.1186/bcr2212 19087323 PMC2656904

[B11] Tamimi RM Eriksson L Lagiou P Czene K Ekbom A Hsieh CC Adami HO Trichopoulos D Hall P Birth weight and mammographic density among postmenopausal women in Sweden Int J Cancer 2010 126 985 991 10.1002/ijc.24786 19642103

[B12] Boyd NF Stone J Martin LJ Jong R Fishell E Yaffe M Hammond G Minkin S The association of breast mitogens with mammographic densities Br J Cancer 2002 87 876 882 10.1038/sj.bjc.6600537 12373602 PMC2376176

[B13] Bentzon N During M Rasmussen BB Mouridsen H Kroman N Prognostic effect of estrogen receptor status across age in primary breast cancer Int J Cancer 2008 122 1089 1094 10.1002/ijc.22892 17960621

[B14] R Development Core Team R. A language and environment for statistical computing 2005 Vienna, Austria: R Foundation for Statistical Computing

[B15] Rogers I The influence of birthweight and intrauterine environment on adiposity and fat distribution in later life Int J Obes Relat Metab Disord 2003 27 755 777 10.1038/sj.ijo.0802316 12821960

[B16] Sachdev HS Fall CH Osmond C Lakshmy R Dey Biswas SK Leary SD Reddy KS Barker DJ Bhargava SK Anthropometric indicators of body composition in young adults: relation to size at birth and serial measurements of body mass index in childhood in the New Delhi birth cohort Am J Clin Nutr 2005 82 456 466 16087993 10.1093/ajcn.82.2.456

[B17] Sellers TA Davis J Cerhan JR Vierkant RA Olson JE Pankratz VS Potter JD Folsom AR Interaction of waist/hip ratio and family history on the risk of hormone receptor-defined breast cancer in a prospective study of postmenopausal women Am J Epidemiol 2002 155 225 233 10.1093/aje/155.3.225 11821247

[B18] Yliharsila H Kajantie E Osmond C Forsen T Barker DJ Eriksson JG Birth size, adult body composition and muscle strength in later life Int J Obes (Lond) 2007 31 1392 1399 10.1038/sj.ijo.0803612 17356523

[B19] Yliharsila H Kajantie E Osmond C Forsen T Barker DJ Eriksson JG Body mass index during childhood and adult body composition in men and women aged 56-70 y Am J Clin Nutr 2008 87 1769 1775 18541567 10.1093/ajcn/87.6.1769

[B20] Friedenreich CM Review of anthropometric factors and breast cancer risk Eur J Cancer Prev 2001 10 15 32 10.1097/00008469-200102000-00003 11263588

[B21] Klein KO Larmore KA de Lancey E Brown JM Considine RV Hassink SG Effect of obesity on estradiol level, and its relationship to leptin, bone maturation, and bone mineral density in children J Clin Endocrinol Metab 1998 83 3469 3475 10.1210/jc.83.10.3469 9768648

[B22] Garnett SP Hogler W Blades B Baur LA Peat J Lee J Cowell CT Relation between hormones and body composition, including bone, in prepubertal children Am J Clin Nutr 2004 80 966 972 15447907 10.1093/ajcn/80.4.966

[B23] Baer HJ Schnitt SJ Connolly JL Byrne C Cho E Willett WC Colditz GA Adolescent diet and incidence of proliferative benign breast disease Cancer Epidemiol Biomarkers Prev 2003 12 1159 1167 14652275

[B24] Emaus A Espetvedt S Veierod MB Ballard-Barbash R Furberg AS Ellison PT Jasienska G Hjartaker A Thune I 17-beta-estradiol in relation to age at menarche and adult obesity in premenopausal women Hum Reprod 2008 23 919 927 10.1093/humrep/dem432 18227106

[B25] Jansen SC Temme EH Schouten EG Lifetime estrogen exposure versus age at menopause as mortality predictor Maturitas 2002 43 105 112 10.1016/S0378-5122(02)00183-4 12385858

[B26] Cooper C Kuh D Egger P Wadsworth M Barker D Childhood growth and age at menarche Br J Obstet Gynaecol 1996 103 814 817 8760713 10.1111/j.1471-0528.1996.tb09879.x

[B27] Terry MB Ferris JS Tehranifar P Wei Y Flom JD Birth weight, postnatal growth, and age at menarche Am J Epidemiol 2009 170 72 79 10.1093/aje/kwp095 19439580 PMC2733039

[B28] Cabanes A Wang M Olivo S DeAssis S Gustafsson JA Khan G Hilakivi-Clarke L Prepubertal estradiol and genistein exposures up-regulate BRCA1 mRNA and reduce mammary tumorigenesis Carcinogenesis 2004 25 741 748 10.1093/carcin/bgh065 14729590

[B29] McCormack VA dos Santos Silva I De Stavola BL Perry N Vinnicombe S Swerdlow AJ Hardy R Kuh D Life-course body size and perimenopausal mammographic parenchymal patterns in the MRC 1946 British birth cohort Br J Cancer 2003 89 852 859 10.1038/sj.bjc.6601207 12942117 PMC2394467

[B30] Hardy DB Janowski BA Chen CC Mendelson CR Progesterone receptor inhibits aromatase and inflammatory response pathways in breast cancer cells via ligand-dependent and ligand-independent mechanisms Mol Endocrinol 2008 22 1812 1824 10.1210/me.2007-0443 18483177 PMC2725768

[B31] Boyd NF Martin LJ Sun L Guo H Chiarelli A Hislop G Yaffe M Minkin S Body size, mammographic density, and breast cancer risk Cancer Epidemiol Biomarkers Prev 2006 15 2086 2092 10.1158/1055-9965.EPI-06-0345 17119032

[B32] Hilakivi-Clarke L Nutritional modulation of terminal end buds: its relevance to breast cancer prevention Curr Cancer Drug Targets 2007 7 465 474 10.2174/156800907781386641 17691906

[B33] De Assis S Hilakivi-Clarke L Timing of dietary estrogenic exposures and breast cancer risk Ann N Y Acad Sci 2006 1089 14 35 10.1196/annals.1386.039 17261753

[B34] Liu S Ginestier C Charafe-Jauffret E Foco H Kleer CG Merajver SD Dontu G Wicha MS BRCA1 regulates human mammary stem/progenitor cell fate Proc Natl Acad Sci USA 2008 105 1680 1685 10.1073/pnas.0711613105 18230721 PMC2234204

[B35] Karp SE Tonin PN Begin LR Martinez JJ Zhang JC Pollak MN Foulkes WD Influence of BRCA1 mutations on nuclear grade and estrogen receptor status of breast carcinoma in Ashkenazi Jewish women Cancer 1997 80 435 441 10.1002/(SICI)1097-0142(19970801)80:3<435::AID-CNCR11>3.0.CO;2-Y 9241077

[B36] Must A Phillips SM Naumova EN Blum M Harris S Dawson-Hughes B Rand WM Recall of early menstrual history and menarcheal body size: after 30 years, how well do women remember? Am J Epidemiol 2002 155 672 679 10.1093/aje/155.7.672 11914195

[B37] Gunnell D Berney L Holland P Maynard M Blane D Frankel S Smith GD How accurately are height, weight and leg length reported by the elderly, and how closely are they related to measurements recorded in childhood? Int J Epidemiol 2000 29 456 464 10.1093/ije/29.3.456 10869317

