# Supplementary material for: Effects of childhood body size on breast cancer tumour characteristics
Source: Breast Cancer Res. 2010 Apr 15;12(2):R23. doi: 10.1186/bcr2564 (PMC2879571; doi:10.1186/bcr2564)
Supplement: Additional file 1 — Table S1. Descriptive characteristics of post-menopausal women. [file bcr2564-S1.DOC]

**Table S**1. Descriptive characteristics of post-menopausal women.

| Characteristic | No. of cases/controls | Cases | Controls | OR* | 95% CI | | P† |
| --- | --- | --- | --- | --- | --- | --- | --- |
|  |  | Mean ± SD | |  |  |  |  |
| Age (years) | 2818/3111 | 63.4±6.7 | 64.3±6.5 | 0.98 | 0.97 | 0.99 | <0.0001 |
| Age at menarche (years) | 2558/2832 | 13.5±1.4 | 13.6±1.4 | 0.96 | 0.93 | 1.00 | 0.0565 |
| Age at menopause (years) | 2803/3093 | 50.4±3.5 | 50.0±3.9 | 1.03 | 1.02 | 1.05 | <0.0001 |
| Parity (No. of live births) | 2818/3110 | 1.8±1.2 | 2.1±1.4 | 0.84 | 0.8 | 0.87 | <0.0001 |
| Age at first birth (years) | 2373/2753 | 25.3±4.9 | 24.6±4.6 | 1.03 | 1.02 | 1.05 | <0.0001 |
| Birth weight (kg) | 1478/1534 | 3.4±0.5 | 3.4±0.5 | 0.96 | 0.83 | 1.1 | 0.5293 |
| Somatotype at age 7 | 2714/2530 | 2.2±1.2 | 2.3±1.3 | 0.87 | 0.8 | 0.95 | 0.001 |
| Somatotype one year prior to enrolment | 2788/2607 | 4.7±1.2 | 4.6±1.2 | 1.04 | 0.94 | 1.15 | 0.1602 |
| Recent body mass index (kg/m^2^) | 2803/3065 | 25.8±4.2 | 25.5±4.2 | 1.02 | 1.01 | 1.03 | 0.0009 |
| Percent mammographic density (%) | 1535/1697 | 17.0±14.1 | 14.1±13.4 | 1.01 | 1.01 | 1.02 | <0.0001 |
|  |  | Percent | |  |  |  |  |
| History of breast cancer in first degree relative (No/Yes) | 2745/2607 | 15.6 | 7.8 | 1.89 | 1.6 | 2.23 | <0.0001 |
| Benign breast disease (Never/Ever) | 2818/3111 | 13.9 | 8.1 | 1.83 | 1.55 | 2.16 | <0.0001 |
| Use of hormone replacement therapy (Never/Ever) | 2811/3087 | 48.4 | 40.3 | 1.34 | 1.21 | 1.49 | <0.0001 |

* Estimated odds ratio and corresponding 95% CI for breast cancer risk adjusted for age at enrolment.

† P-values based on Wald tests. All logistic regression models adjusted for age at enrolment.
