# Supplementary material for: Effects of childhood body size on breast cancer tumour characteristics
Source: Breast Cancer Res. 2010 Apr 15;12(2):R23. doi: 10.1186/bcr2564 (PMC2879571; doi:10.1186/bcr2564)
Supplement: Additional file 2 — Table S2. Multivariate-adjusted odds ratio (OR) estimates and corresponding 95% confidence intervals (CIs) of postmenopausal breast cancer for somatotype at age seven years on a subset of women with mammographic density data; overall and stratified by breast cancer tumour subtype based on estrogen receptor (ER) and progesterone receptor (PR) status. [file bcr2564-S2.DOC]

**Table S2. Multivariate-adjusted OR estimates and corresponding 95% CIs of postmenopausal breast cancer for somatotype at age 7 on a** subset of women with mammographic density data; overall and stratified by breast cancer tumour subtype based on ER and PR status.

| Type of breast cancer | Somatotype | Without adjustment for MD | | | | |  | With adjustment for MD | | | | |
| --- | --- | --- | --- | --- | --- | --- | --- | --- | --- | --- | --- | --- |
|  |  | Cases | OR | 95% CI | | P trend* |  | Cases | OR | 95% CI | | P trend† |
| All data | Lean | 902 | 1.00 | reference | | 0.001 |  | 902 | 1.00 | reference | | 0.001 |
|  | Medium | 353 | 0.79 | 0.66 | 0.94 |  |  | 353 | 0.79 | 0.66 | 0.94 |  |
|  | Large | 79 | 0.66 | 0.48 | 0.90 |  |  | 79 | 0.67 | 0.49 | 0.92 |  |
| ER-positive | Lean | 510 | 1.00 | reference | | 0.012 |  | 510 | 1.00 | reference | | 0.019 |
|  | Medium | 200 | 0.79 | 0.65 | 0.98 |  |  | 200 | 0.80 | 0.65 | 0.98 |  |
|  | Large | 49 | 0.73 | 0.50 | 1.04 |  |  | 49 | 0.75 | 0.52 | 1.08 |  |
| ER-negative | Lean | 100 | 1.00 | reference | | 0.004 |  | 100 | 1.00 | reference | | 0.006 |
|  | Medium | 34 | 0.66 | 0.43 | 0.99 |  |  | 34 | 0.66 | 0.44 | 1.01 |  |
|  | Large | 5 | 0.34 | 0.14 | 0.87 |  |  | 5 | 0.36 | 0.14 | 0.90 |  |
| PR-positive | Lean | 445 | 1.00 | reference | | 0.012 |  | 445 | 1.00 | reference | | 0.018 |
|  | Medium | 170 | 0.77 | 0.62 | 0.95 |  |  | 170 | 0.77 | 0.62 | 0.96 |  |
|  | Large | 44 | 0.73 | 0.50 | 1.07 |  |  | 44 | 0.76 | 0.52 | 1.12 |  |
| PR-negative | Lean | 155 | 1.00 | reference | | 0.008 |  | 155 | 1.00 | reference | | 0.010 |
|  | Medium | 59 | 0.77 | 0.55 | 1.06 |  |  | 59 | 0.77 | 0.56 | 1.07 |  |
|  | Large | 9 | 0.43 | 0.21 | 0.87 |  |  | 9 | 0.44 | 0.21 | 0.89 |  |
| * Logistic regression models were used, accounting for age, age at menarche, benign breast disease and recent body mass index. | | | | | | | | | | | | |
| † Logistic regression models were used, accounting for the same variables in the previous model and in addition, mammographic density using a subset of the women with percent density data available (N=3232). | | | | | | | | | | | | |
